# The Use of Pepto Mangan for Anæmia in Pulmonary Tuberculosis

**Published:** 1895-03

**Authors:** Karl Von Ruck

**Affiliations:** Asheville, N. C., Director of the Winyah Sanitarium for Diseases of the Lungs and Throat


### THE USE OF PEPTO-MANGAN FOR ANÆMIA IN PULMONARY TUBERCULOSIS.

BY KARL VON RUCK, B. S., M. D., ASHEVILLE, N. C.,
Director of the Winyah Sanitarium for Diseases of the Lungs and Throat.

(*Reprint from the New York Medical Journal, Dec. 15, 1894.*)

The anæmia of tuberculosis differs from some other forms in being, as a rule, the result of the deleterious effects of toxine upon the blood, or upon the blood-making organs.

Simple anæmia at times precedes the development of tuberculosis, and becomes a predisposing factor to infection with the specific germs, and in the course of the disease such anæmic states may also result from gastro-intestinal complications. These do not come within the limits of this paper. While the toxic form can not always be distinguished because frequently associated with the other, toxines must be recognized, nevertheless, as a chief cause of anæmia in all contagious and infectious diseases. In its treatment the indication is, of course, the prevention of the production of toxines within the body, which can only be accomplished by the removal of the pathogenic germs or by the production of immunity from their toxines.

The destruction of the specific germs of tuberculosis within the living organisms, or immunity from their toxic products, occur naturally in strong and healthy persons who show no predisposition to the acquirement of tuberculosis. If infection occurs in such, their tissues, and especially the blood, are capable of offering successful resistance, and the organism is preserved in its integrity.

In the established disease, the resisting power of the particular patient has evidently been insufficient, either by reason of the organism being overpowered by the excessive quantity of infecting material, or by reason of the minor resistance on the part of the tissues where the specific germs gained entrance. This view is so uniformly accepted, and in the light of pathological investigation as well as of clinical experience it is so well proved, that there is no necessity for me to dwell upon it further, neither is its elucidation contemplated in this paper.

While our direct treatment of the disease with specific germicidal remedies, inaugurated by Professor Koch four years ago, is now still further advanced toward success through the purification of the remedy by Professor Edwin Klebs, and by the experience obtained since its introduction, we must, nevertheless, not lose sight of the resisting power of the patient and of its increase; as far as that may be possible, it must be accomplished, if we expect to deal most successfully with the disease. In the application of the specific culture products in pulmonary tuberculosis my observations have amply confirmed this view, and the best results are being obtained in cases where the general strength of the patient is still good, and especially when the blood approaches a normal standard in corpuscles and hæmoglobin.

Most tubercular patients show a considerable loss in these respects, even in the early stages, and these losses I have often observed to progress despite a good appetite, and in patients who for the time gained in weight. That the anæmia in such cases is of toxic origin is proved by the fact that the losses become balanced under specific treatment, and my records show many instances in which the administration of tuberculin, and more particularly larger doses of antiphthisin (Klebs), showed that a slow regeneration of the blood followed their use, while the febrile movement accompanying the resorption of toxines disappeared.

It is therefore quite rational to seek to aid the regeneration of the blood, the more so as in all advanced cases it takes place very slowly if at all; and in addition to proper dietetic and hygienic management one looks naturally to ferruginous remedies for its accomplishment. This has been my endeavor under every method of treatment, but the available preparations have not only failed me in the majority of instances, but they have frequently interfered with the improvement in other respects by disturbing the digestion and assimilation of food. After many more or less indifferent results, I came to discard iron in all pharmaceutical forms, and resorted to rectal injections of defibrinated blood, which, although very inconvenient and frequently objectionable to the patient, accomplished my object most satisfactorily until some twelve months ago, when I rather reluctantly undertook the administration of PEPTO-MANGAN (Gude's), chiefly because some of my patients positively refused the blood injections, and because of the laudation of the remedy in German medical literature by authorities personally known to me to be reliable.

In its clinical use I found, in the first place, that the remedy was palatable and readily taken by the patients, and from its first use to the present time I have had only two cases in which I was obliged to abandon it. These were cases with tubercular ulceration of the epiglottis, and they complained of more smarting pain and distress than from the swallowing of ordinary liquids and foods, even when the remedy was largely diluted; in all other cases it was well borne by the stomach; in quite a number the appetite improved very early; neither is there evidence of its producing constipation in any case.

It would extend this paper beyond reasonable limits to give the details of the comparative examinations of the blood made from time to time in upwards of seventy patients who received this remedy; suffice it to say that the most improved instruments and apparatus were used, and that all sources of error were carefully excluded. The results in the first series of experiments (comparing twelve patients) were as follows:

COMPARATIVE TABLE FOR TWELVE PATIENTS TREATED WITH PEPTO-MANGAN (Gude's) SIX WEEKS.

| | In six weeks previous the patient had | | | | At beginning of PEPTO-MANGAN (Gude's). | | After six weeks' use of PEPTO-MANGAN (Gude's). | | Under PEPTO-MANGAN (Gude's) the patient had | | | |
|---|---|---|---|---|---|---|---|---|---|---|---|---|
| | Lost. | | Gained. | | | | | | Lost. | | Gained. | |
| | Number of corpuscles. | Hæmoglobin, per ct. | Number of corpuscles. | Hæmoglobin, per ct. | Number of corpuscles. | Hæmoglobin, per ct. | Number of corpuscles. | Hæmoglobin, per ct. | Number of corpuscles. | Hæmoglobin, per ct. | Number of corpuscles. | Hæmoglobin, per ct. |
| 1. | .... | .... | 141,000 | 4 | 3,400,000 | 72 | 3,740,000 | 83 | .... | .... | 340,000 | 11 |
| 2. | .... | .... | 88,000 | 3 | 2,716,000 | 58 | 3,390,000 | 76 | .... | .... | 674,000 | 18 |
| 3. | .... | 2 | 14,000 | .... | 3,088,000 | 74 | 3,176,000 | 79 | .... | .... | 78,000 | 5 |
| 4.* | 740,000 | 9 | .... | .... | 2,015,000 | 42 | 1,792,000 | 40 | 223,000 | 2 | .... | .... |
| 5. | .... | .... | 166,000 | 2 | 3,853,000 | 85 | 4,600,000 | 92 | .... | .... | 747,000 | 7 |
| 6.† | 301,800 | 7 | .... | .... | 2,610,000 | 59 | 3,543,000 | 88 | .... | .... | 933,000 | 29 |
| 7.‡ | .... | .... | .... | .... | 1,807,000 | 33 | 3,600,000 | 69 | .... | .... | 1,793,000 | 36 |
| 8. | .... | .... | 390,000 | 4 | 4,389,000 | 81 | 4,422,000 | 92 | .... | .... | 33,000 | 11 |
| 9.§ | .... | .... | 94,000 | 10 | 3,700,000 | 66 | 4,800,000 | 97 | .... | .... | 1,100,000 | 31 |
| 10.‖ | 511,000 | 13 | .... | .... | 2,005,000 | 49 | 3,983,000 | 90 | .... | .... | 1,978,000 | 41 |
| 11.¶ | .... | .... | .... | .... | 1,547,000 | 27 | 3,545,000 | 73 | .... | .... | 1,992,000 | 46 |
| 12. | .... | .... | 815,000 | 30 | 4,002,000 | 90 | 4,612,000 | 93 | .... | .... | 610,000 | 3 |

* Patient suffered from advanced pulmonary tuberculosis with occasional colliquative diarrhœa, and had lost in corpuscles 740,000 and hæmoglobin nine per cent. in the four weeks preceding.

† Patient had toxic temperature in a mild degree, and had lost 301,800 corpuscles and seven per cent. of hæmoglobin in six weeks previous; did not receive any specific treatment.

‡ Patient also received tuberculocidin (Klebs), under which the temperature became normal in the third week; only recently admitted; no previous examination for comparison.

§ Early-stage case; received tuberculocidin (Klebs), 0.01 to 3 cubic centimetres, under which tubercle bacilli disappeared from the sputum and physical signs were no longer demonstrable.

‖ Early-stage case; gastric catarrh; stomach was washed every day for three weeks, then occasionally.

¶ Patient just admitted; a month previous suffered from pulmonary hæmorrhage, with acute tubercular pneumonia following; received tuberculocidin (Klebs), 0.005 to 2.4—in all, 21 cubic centimetres; *gained eleven pounds in weight.*

If we now examine this table, we find that in the six weeks previous to the use of the PEPTO-MANGAN (Gude's), we have ten patients in whom the loss or gain in their blood condition could be compared; seven of these patients gained in all 1,408,-000, or an average of 200,000 each, whereas these same patients gained under PEPTO-MANGAN (Gude's), 3,609,000 corpuscles, or an average of 510,000 for each.

As to hæmoglobin, a similar increase is perceptible. In six weeks preceding, of the seven patients, six also gained in hæmoglobin in all fifty-six per cent., or an average of nine per cent.; but under PEPTO-MANGAN (Gude's) these same six patients gained in all eighty-one per cent., and on an average thirteen and a half per cent.

Further, whereas of the ten patients only seven gained in corpuscles and six in hæmoglobin in the six weeks preceding, under the PEPTO-MANGAN (Gude's), nine gained in corpuscles and hæmoglobin, and no loss occurred except in one, and she lost only a third as much as in the six weeks before. Cases VI. and X. are particularly to be noticed. In Case X., the patient, having suffered a loss of half a million corpuscles and thirteen per cent. of hæmoglobin, gained in the six weeks' treatment with PEPTO-MANGAN (Gude's), two million corpuscles and forty-one per cent. of hæmoglobin. It is, however, true that he also received local treatment for his gastric catarrh; but that treatment was applied during three of the previous six weeks without being able to check the rapid loss, In Case VI., the patient, having previously lost 301,800 corpuscles and seven per cent. hæmoglobin, gained 933,000 corpuscles and twenty-nine per cent. hæmoglobin.

When these comparisons were completed, examinations were made at less frequent intervals, and the use of PEPTO-MANGAN (Gude's) was more generally adopted in my institution.

Another series of cases was more accurately observed within the last six months, and the results were practically the same as in the table. In all cases the improvement of the blood conditions was highly satisfactory; in quite a number phenomenal. The degeneration, fragmentation, and disappearance of the tubercle bacilli from the patient's sputum, while heretofore also observed under tuberculin, and more particularly under antiphthisin (Klebs), was certainly more rapid in the cases in which the blood examinations showed rapid improvement—to my way of thinking, a good proof of the germicidal action of the blood, and of the great importance of improving its condition whenever any impairment becomes manifest.

As to the superiority of this particular "iron and manganese" preparation over others, and as to the value of the manganese in the combination, I will not pretend to offer any explanations. I simply wish to record clinical facts, in the belief that all practitioners will gladly welcome any remedy which can so apparently aid in the anæmia of tuberculosis, as appears from the preceding table, and in other anæmic states, as shown from the reports of Dr. H. P. Loomis and others.

